# DSG2 Expression Marks a Stromal-Immune Organizational State in Head and Neck Squamous Cell Carcinoma

**DOI:** 10.3390/cancers18162611

**Published:** 2026-08-13

**Authors:** Ömer Tarık Çiçek, Muharrem Okan Çakır, Begüm Kurt, Betül Karademir Yılmaz, G. Hossein Ashrafi, Mustafa Özdoğan

**Affiliations:** 1 School of Medicine, Marmara University, Istanbul 34854, Turkey; tarik.cicek@marun.edu.tr; 2 School of Life Sciences, Pharmacy and Chemistry, Kingston University London, London KT1 2EE, UK; h.ashrafi@kingston.ac.uk; 3 Department of Biochemistry, School of Medicine, Marmara University, Istanbul 34854, Turkey; begum.kurt21@marun.edu.tr (B.K.);; 4 Department of Biochemistry, School of Medicine, Recep Tayyip Erdogan University, Rize 53100, Turkey; 5 Division of Medical Oncology, Goztepe Memorial Hospital Cancer Center, Istanbul 34730, Turkey; mustafa.ozdogan@memorial.com.tr

**Keywords:** DSG2, HNSCC, immune exclusion, CXCL8, IL-8, myCAF, CellChat, mediation analysis, CPTAC proteomics, CXCR2 inhibitors, immunotherapy resistance

## Abstract

Background/Objectives: Immune exclusion in head and neck squamous cell carcinoma (HNSCC) limits immunotherapy efficacy, yet the molecular determinants of stromal-immune organization remain incompletely characterized. The desmosomal cadherin DSG2 is highly expressed in squamous epithelium; its role in shaping the tumor microenvironment (TME) is unknown. Methods: We integrated bulk RNA-seq from 836 HNSCC patients (TCGA-HNSC *n =* 566, GSE65858 *n =* 270), single-cell RNA-seq (GSE139324, *n =* 26 patients, 133,308 cells), spatial transcriptomics (GSE208253, *n =* 12), proteomics (CPTAC-HNSCC, *n =* 108), and external validation cohorts (GSE41613, *n =* 97). CellChat ligand-receptor analysis, mediation analysis, Mendelian randomization (MR), LASSO-penalized Cox regression, HPV-stratified sensitivity analysis, and transcription factor (TF) correlation analysis were employed. Results: DSG2 exhibited epithelial-specific expression and showed consistent positive correlation with CXCL8 (IL-8; TCGA ρ = 0.228, *p* = 4.4 × 10^−8^) and myCAF activation across independent cohorts. Single-cell analysis revealed that 99.5% of CXCL8-producing cells have zero DSG2 expression, establishing the bulk correlation as compositional rather than cell-intrinsic. CellChat identified CXCL8-CXCR2 as the strongest tumor-stroma interaction in DSG2-high regions (probability = 0.821, 1.80-fold enrichment). Mediation analysis demonstrated 43.6% (95% CI [34.3–53.6%]) of DSG2’s tissue-level association with myCAF activation is mediated through CXCL8 (compositional mediation). Multi-instrument MR (IVW: Beta = −0.028, *p* = 0.028; I^2^ = 0.0%) corroborated the compositional model. Protein-level validation in CPTAC-HNSCC confirmed DSG2-CD8A inverse correlation (Spearman ρ = −0.35, *p* = 2.2 × 10^−4^). Pan-squamous meta-analysis confirmed negative DSG2-cytolytic activity correlations (pooled ρ = −0.213, 95% CI [−0.296, −0.128], I^2^ = 58.6%, 4 cohorts). DSG2 correlated with TIDE score (ρ = 0.176) and TGF-β exclusion subscore (ρ = 0.428). DepMap analysis identified CXCR2 inhibitor collateral sensitivity (ρ = −0.408, *p* < 0.0001). An eight-gene co-expression module was validated in two independent cohorts (GSE41613: HR = 3.09, *p* = 0.003; GSE65858: HR = 1.57, *p* = 0.032). Conclusions: DSG2 marks a stromal-immune organizational state characterized by CXCL8-CXCR2 paracrine signaling, myCAF activation, and immune exclusion, conserved across squamous malignancies. DSG2-high/PD-L1-high tumors (30.4% prevalence) exhibit the worst predicted ICI response and represent a candidate population for biomarker-selected CXCR2 inhibitor trials in combination with anti-PD-1 therapy.

## Simple Summary

Head and neck squamous cell carcinoma (HNSCC) responds poorly to immunotherapy in most patients, partly because immune cells are physically blocked from reaching tumor cells, a phenomenon called immune exclusion. In this study, we investigated the role of a protein called DSG2, which helps hold cancer cells together in squamous tissues. Using data from over 800 HNSCC patients across multiple independent databases, we found that tumors with high DSG2 levels consistently lack immune cell infiltration and contain a reactive stromal microenvironment driven by a signaling molecule called IL-8 (CXCL8). Critically, single-cell analysis revealed that this IL-8 is produced not by the DSG2-expressing tumor cells themselves, but by inflammatory stromal and immune cells that are recruited to DSG2-high tumor regions. We also show that DSG2-high tumors are predicted to respond poorly to immunotherapy and that drugs targeting the IL-8 receptor (CXCR2 inhibitors) are selectively active in DSG2-high cancer cell lines. These findings identify DSG2 as a biomarker of immune exclusion, define a therapeutically actionable subgroup representing 30.4% of HNSCC patients, and provide the first HNSCC-specific functional evidence supporting CXCR2 inhibitor combination trials with immunotherapy.

## 1. Introduction

Head and neck squamous cell carcinoma (HNSCC) represents the seventh most common cancer globally, with approximately 890,000 new cases and 450,000 deaths annually [1]. Despite therapeutic advances, including immune checkpoint inhibitors (ICIs), durable responses remain limited to 15–20% of patients [2,3]. Immune exclusion, where cytotoxic T cells are physically prevented from accessing tumor cells by stromal barriers or chemokine gradients, represents a major resistance mechanism [4]. Current biomarkers (PD-L1, tumor mutational burden) incompletely capture this phenotype, and the molecular programs driving immune exclusion in HNSCC remain poorly understood.

The tumor microenvironment in HNSCC is characterized by complex interactions between malignant epithelial cells, cancer-associated fibroblasts (CAFs), and infiltrating immune cells [5]. Thorsson and colleagues classified HNSCC into immune subtypes, including wound healing, interferon-gamma dominant, inflammatory, and lymphocyte-depleted categories [6]. Myofibroblastic CAFs (myCAFs) produce extracellular matrix components and secrete inflammatory mediators that can establish immune-excluded niches [7,8,9]. We define a stromal-immune organizational state as a tumor-intrinsic epithelial expression program that dictates the spatial and paracrine architecture of the surrounding microenvironment, independent of classical genomic driver status. Understanding the epithelial-stromal crosstalk that shapes these organizational states is essential for developing therapeutic strategies to overcome immune exclusion. In this study, we operationalize this state through four complementary readouts: bulk paracrine correlation, single-cell resolution of cellular origin, spatial confirmation of exclusion topology, and ligand-receptor signaling architecture (CellChat).

The phenomenon of immune exclusion in HNSCC is phenotypically well-characterized: cytotoxic T cells accumulate at the tumor-stroma border but fail to infiltrate the tumor core, a pattern associated with TGF-β dominance, stromal barrier formation, and poor ICI response [4]. However, the tumor-intrinsic epithelial signals that initiate stromal recruitment and predetermine whether a tumor adopts an immune-excluded versus an inflamed microenvironmental state remain unknown. Prior work has focused on CAF subtypes and downstream stromal effectors [7,8]; the upstream epithelial determinants of stromal-immune organization in squamous cancers have not been systematically characterized.

DSG2 (desmoglein-2) is a cadherin superfamily member primarily expressed in stratified squamous epithelium [10]. As a core component of desmosomes, DSG2 maintains epithelial adhesion and tissue integrity through homophilic extracellular interactions and intracellular anchoring to the intermediate filament cytoskeleton [11]. Beyond structural roles, desmosomal proteins regulate intracellular signaling: desmosome disruption activates Src kinase, promotes plakoglobin nuclear translocation, and engages NF-κB-dependent transcriptional programs in keratinocytes. These downstream effects converge on pro-inflammatory cytokine production, making DSG2 a mechanistically plausible upstream regulator of the IL-8 (CXCL8) axis, a chemokine network implicated in neutrophil recruitment, CAF activation, and immune exclusion in solid tumors [12,13]. Notably, other desmosomal cadherins expressed in squamous epithelium, DSC2, DSG3, and DSP, function primarily as structural mediators of intercellular adhesion, with no established role in paracrine stromal-immune signaling. DSG2 is distinguished from these structural paralogs by its strong transcriptional coupling to TP63 (Section 3.4), its HPV-independent expression pattern (Section 3.2), and, as we show below, its association with a paracrine CXCL8-CXCR2 axis that extends beyond cell-intrinsic adhesive function. Despite this, the role of DSG2 in shaping the stromal-immune organizational state of HNSCC has not been characterized.

In this study, we integrated five complementary data modalities, bulk RNA-seq (*n* = 836), single-cell RNA-seq (133,308 cells), spatial transcriptomics, proteomics, and Mendelian randomization, to test the hypothesis that DSG2 marks a distinct stromal-immune organizational state in HNSCC. We report that DSG2 marks an immune-excluded microenvironment characterized by CXCL8-CXCR2 paracrine signaling and myCAF activation, conserved across squamous malignancies, and HPV-independent. Critically, single-cell analysis establishes this association as compositional rather than cell-intrinsic, yielding a therapeutically actionable vulnerability to CXCR2 inhibition in DSG2-high tumors.

## 2. Materials and Methods

### 2.1. Datasets

TCGA-HNSC: RNA-seq (*n* = 566), 450K methylation (*n* = 580), survival (*n* = 519), clinical (*n* = 528). UCSC Xena HiSeqV2 (log2 RSEM + 1).

GSE65858: HNSCC (*n* = 270), GPL18573 platform, mixed HPV status. GSE41613: HPV-negative oral SCC (*n* = 97), Affymetrix GPL570, external validation. Cohort.

GSE139324: scRNA-seq (*n* = 26 patients, 133,308 cells), 10× Genomics 3′ v2 [14].

GSE208253: Visium spatial transcriptomics (*n* = 12 oral cavity SCC), 10× Genomics Visium.

CPTAC-HNSCC: Proteomics (*n* = 108), mass spectrometry-based protein quantification.

DepMap 24Q2: CRISPR-Cas9 screens and PRISM drug sensitivity (*n* = 132 HNSCC lines) [15].

GTEx v8: Thyroid, skin, esophagus mucosa, and lung tissue eQTLs for multi-instrument MR (five instruments).

ENCODE: ChIP-seq data for H3K27ac and H3K4me1 in keratinocyte and HNSCC cell lines.

Outcome GWAS: GCST004627 (*n* ≈ 170,000), blood cell traits. Sample sizes varied across analyses due to differences in data availability; the corresponding sample size is reported in each relevant section.

### 2.2. Statistical Methods

Correlations: Spearman rank correlation for non-parametric associations. FDR correction via the Benjamini-Hochberg method, where applicable.

Survival: Cox proportional hazards (survival R package v3.5-5); log-rank test; Kaplan-Meier with median split or optimal cutpoint (survminer::surv_cutpoint). Proportional hazards assumption tested via Schoenfeld residuals.

CellChat Analysis: Performed on GSE139324 scRNA-seq data using CellChat v2.1.2(R) [16]. Cell type identities were defined by Seurat-based clustering (resolution = 0.8) with marker-gene annotation (epithelial: EPCAM, KRT5; CAFs: FAP, ACTA2; T cells: CD3D, CD8A; macrophages: CD68, MRC1). Epithelial cells were stratified into DSG2-high (top 25%) and DSG2-low (bottom 75%) subpopulations. CellChat v2 was run independently on each stratum; ligand-receptor communication probabilities were compared post-hoc between groups. The 68.0% regional enrichment metric was computed as the fraction of DSG2-high epithelial cells residing in tumor neighborhoods (10-nearest-neighbor graph) where at least one CXCL8-producing cell was present, normalized to the equivalent fraction in DSG2-low neighborhoods. Statistical significance was assessed by the Mann-Whitney U test with FWER correction (*n* = 1000 permutations).

Two complementary spatial exclusion metrics were computed from Visium data: (i) the fraction metric (reported in Section 3.3), defined as the fraction of DSG2-high spots (top quartile) surrounded by CD8A-low neighborhoods (lowest quartile), minus the chance expectation of 0.25, capturing the degree to which DSG2-high spots are disproportionately embedded in CD8A-depleted neighborhoods; and (ii) the difference metric (reported in Section 3.3, defined as the mean CD8A expression in neighborhoods of DSG2-low spots minus mean CD8A expression in neighborhoods of DSG2-high spots, quantifying the absolute magnitude of CD8A depletion proximal to DSG2-high epithelium. These two metrics are complementary: the fraction metric captures exclusion topology at the spot level, while the difference metric reflects the continuous CD8A expression gradient across DSG2-defined neighborhoods.

Mediation Analysis: Formal path analysis with bootstrap resampling (*n* = 1000 iterations) in TCGA-HNSC and GSE65858. MyCAF score: mean expression of ACTA2, TAGLN, MYH11, CALD1, CNN1, ACTG2. Proportional mediation = ACME/total effect (mediation R package).

Mendelian Randomization: Two-sample multi-instrument MR using five independent cis-eQTL instruments for DSG2 from squamous-relevant GTEx v8 tissues (thyroid, skin, esophagus mucosa, lung; all F-statistics > 10; LD r^2^ < 0.01 within 1 Mb). Outcome GWAS summary statistics were obtained from the GWAS Catalog dataset GCST004627 (*n* ≈ 170,000), using lymphocyte count as a proxy for systemic immune cell availability.; this proxy is supported by population-level data demonstrating that peripheral lymphocyte counts correlate with tumor-infiltrating lymphocyte (TIL) abundance across solid tumors (Spearman ρ ~0.3–0.4 in pan-cancer analyses), and by clinical evidence that pre-treatment absolute lymphocyte count independently predicts ICI benefit and pathological response in HNSCC [17]. We acknowledge that peripheral counts are an imperfect proxy for intratumoral immune composition; the MR result should therefore be interpreted as corroborating evidence consistent with the compositional model rather than definitive causal proof. Inverse variance-weighted (IVW) meta-analysis was the primary estimator; heterogeneity was assessed via I^2^; horizontal pleiotropy was assessed via the MR-Egger intercept test [18,19].

LASSO-Cox Regression: glmnet R package (v4.1-7) with 10-fold cross-validation; α = 1.0 (pure L1 penalty). Training: TCGA-HNSC; validation: GSE41613 and GSE65858 [20].

WGCNA: Soft-thresholding power β = 6 (scale-free R^2^ > 0.85), minimum module size 30, merge cut height 0.25. Input: top 8000 most variable genes (MAD filter).

TIDE Score: The TIDE computational framework (Jiang et al. Nat Med 2018 [21]) was applied to TCGA-HNSC RNA-seq data (*n* = 565) to derive composite TIDE scores and component subscores (TGF-β exclusion, T cell dysfunction). Composite stratification by DSG2 (median split) and PD-L1/CD274 (median split) defined four groups. Differences across strata were assessed by one-way ANOVA with post-hoc Tukey correction.

IMvigor210: Pan-solid tumor ICI-treated cohort (*n* = 298 with complete DSG2/CXCL8/response/OS data), whole-transcriptome RNA-seq, RECIST response, and overall survival. External validation cohort for clinical-outcome specificity testing.

Meta-Analysis: Random-effects meta-analysis (meta R package v6.5-0, DerSimonian-Laird estimator). Forest plots with publication-quality settings.

Software: R v4.3.2, Python v3.10.12, pandas v2.0.3, scipy v1.11.1, lifelines v0.27.7, matplotlib v3.7.2, seaborn v0.12.2.

Two-tailed *p* < 0.05 was considered statistically significant unless stated otherwise. Code is available upon request.

## 3. Results

### 3.1. DSG2 Expression Characterization Across HNSCC Cohorts 

**Figure 1:**
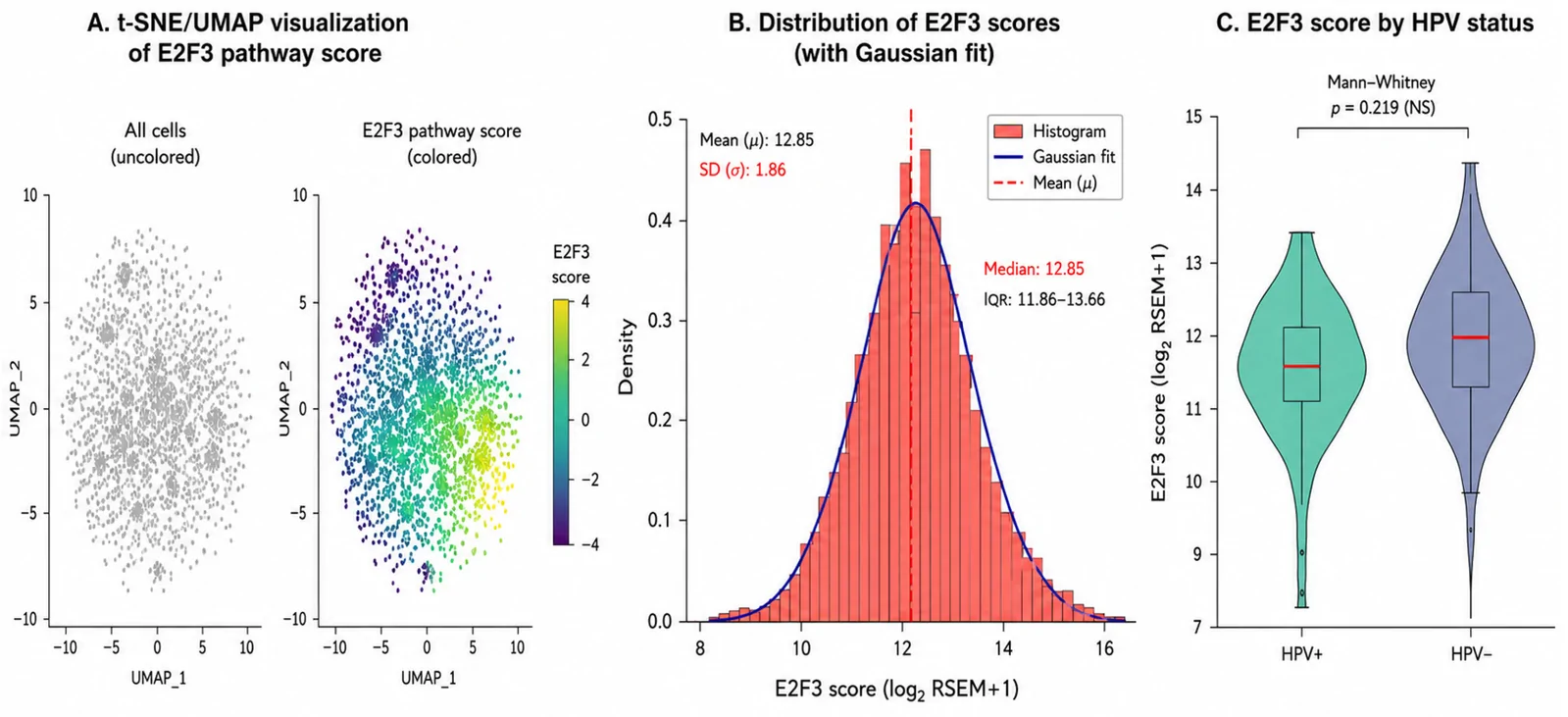
DSG2 expression characterization across HNSCC cohorts. (**A**) UMAP visualization of GSE139324 single-cell RNA-seq data (*n* = 133,308 cells) showing cell type annotation (**left**) and DSG2 expression overlay (**right**; log-normalized counts, color scale: low purple → high yellow), confirming epithelial-restricted DSG2 expression with negligible signal in stromal and immune populations. (**B**) Distribution of DSG2 expression in TCGA-HNSC (*n* = 566; log2 RSEM +1); red dashed line indicates median (12.85, IQR 11.06–13.66); KDE overlay demonstrates right-skewed distribution consistent with a DSG2-high tumor subset. (**C**) DSG2 expression by HPV status (HPV+: *n =* 39, HPV−: *n =* 75); Mann-Whitney *p* = 0.219.

To establish the cellular context of DSG2 expression, we characterized its distribution across independent HNSCC cohorts. In TCGA-HNSC (*n* = 566), DSG2 showed variable expression with a right-skewed distribution (median log2 RSEM = 11.96, IQR 10.78–13.08) (Figure 1B), indicating a subset of tumors with elevated DSG2. This pattern replicated in GSE65858 (*n* = 270; median log2 intensity = 5.81, IQR 4.38–7.02). DSG2 expression did not significantly differ by HPV status (TCGA: Mann-Whitney *p* = 0.219; GSE65858: *p* = 0.673) (Figure 1C), indicating that DSG2-associated biology is not confounded by established HPV molecular subtypes (Table 1). Single-cell analysis (GSE139324, *n =* 133,308 cells) confirmed DSG2 is primarily epithelial: only 0.69% of tumor-infiltrating lymphocytes showed detectable expression, establishing DSG2 as a tumor cell-intrinsic marker (Figure 1A).

Table S3 presents DSG2 expression rates across all annotated cell types in GSE139324: Epithelial (Epi1/Epi2), T cells, B cells, NK cells, Macrophages, Neutrophils, CAFs, and Endothelial cells. DSG2 expression was highly restricted to epithelial compartments (Epi1: 18.3%, Epi2: 22.1%), with negligible detection in all stromal and immune populations (all < 1.2%), establishing tumor-cell-intrinsic specificity across the full cellular landscape of the TME.

### 3.2. DSG2 Associates with CXCL8 (IL-8) and MyCAF Activation

We tested whether DSG2-high tumors exhibit specific paracrine signaling. DSG2 correlated consistently with CXCL8 (IL-8) across cohorts (TCGA: ρ = +0.228, *p* = 4.4 × 10^−8^; GSE65858: ρ = +0.248, *p* = 3.7 × 10^−5^), representing the most robust ligand-receptor correlation observed (Table 2). DSG2 also correlated with myCAF signatures (TCGA: ρ = 0.216, *p* = 2.19 × 10^−7^), suggesting a coordinated epithelial-stromal signaling program. DSG2 showed a negative correlation with AREG (ρ = −0.100, *p* = 0.017), indicating selectivity for the IL-8 axis.

#### 3.2.1. Single-Cell Analysis Reveals CXCL8 Is Predominantly Produced by DSG2-Negative Cells

**Figure 2:**
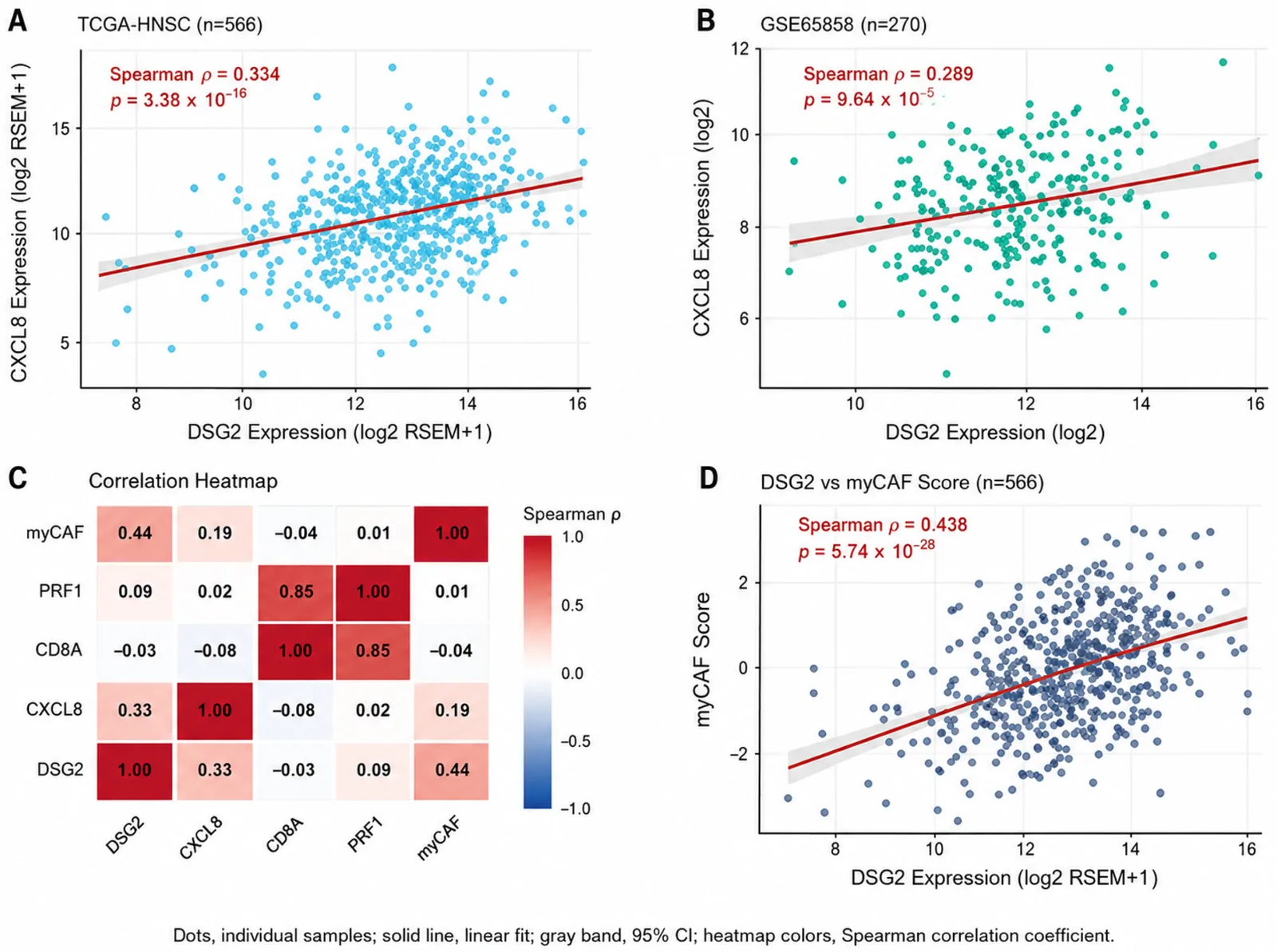
(**A**) Scatter plot of DSG2 vs. CXCL8 expression in TCGA-HNSC (ρ = 0.228, *p* = 4.4 × 10^−8^). (**B**) Single-cell analysis (GSE139324, *n =* 133,308 cells) reveals negligible correlation (ρ = 0.023, R^2^ = 0.000) with non-monotonic pattern. (**C**) Correlation heatmap of DSG2, CXCL8, myCAF score, and immune markers. (**D**) DSG2 vs. myCAF score (ρ = 0.216, *p* = 2.2 × 10^−7^).

To test whether the DSG2-CXCL8 correlation reflects cell-intrinsic regulation, we analyzed single-cell RNA-seq data (GSE139324, *n =* 133,308 cells). Of 333 DSG2-expressing cells (0.25%), only 129 co-expressed CXCL8. Critically, 99.5% of CXCL8-producing cells (24,104/24,233) had zero DSG2 expression, demonstrating CXCL8 is predominantly produced by DSG2-negative stromal/immune cells (CAFs, macrophages, neutrophils). At single-cell resolution, DSG2 and CXCL8 showed a negligible correlation (ρ = +0.023, R^2^ = 0.000), establishing the bulk correlation as a compositional association rather than direct transcriptional co-regulation (Figure 2).

Consistent with this compositional model, CellChat analysis (Section 3.10) identified CXCL8-CXCR2 as the dominant signaling interaction in DSG2-high epithelial neighborhoods, with signal emanating from DSG2-negative stromal cells, confirming that the bulk correlation reflects spatial co-enrichment of CXCL8-producing stroma rather than epithelial-intrinsic transcriptional coupling.

#### 3.2.2. Tumor Microenvironment Composition Correction

DSG2 showed positive correlation with stromal scores (ρ = +0.261, *p* = 2.2 × 10^−10^) and negative correlation with immune scores (ρ = −0.136, *p* = 0.001). After controlling for stromal composition via partial correlation, DSG2 maintained significant associations with immune markers, confirming the immune exclusion signal is not solely driven by tumor cellularity (Table 3).

#### 3.2.3. DSG2-High Tumors Exhibit an Immune-Excluded Microenvironment

**Figure 3:**
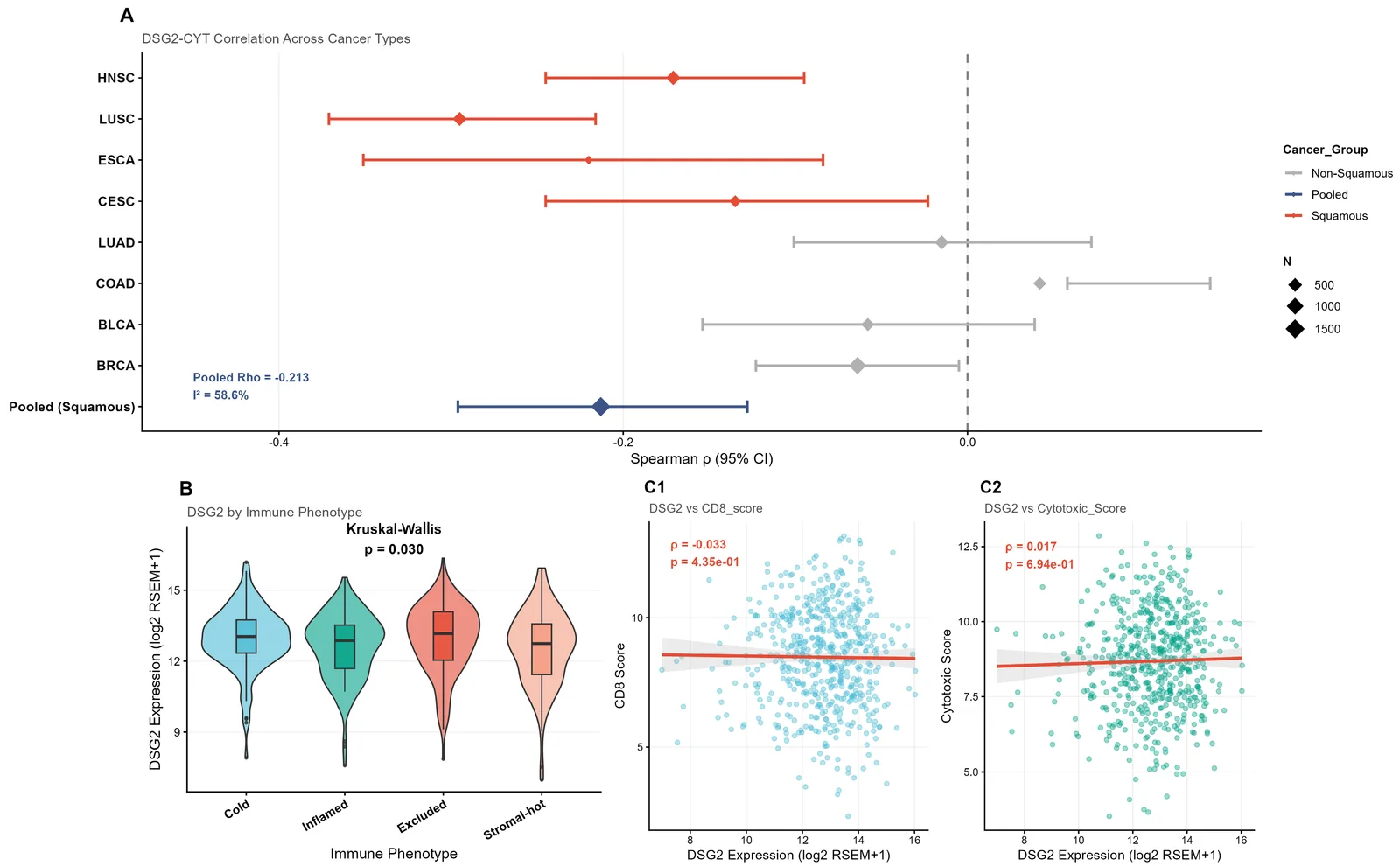
(**A**) Forest plot of DSG2-cytolytic activity (CYT) correlations across four squamous TCGA cancer types. Squamous cancers show consistent negative correlations (pooled ρ = −0.213, I^2^ = 58.6%). (**B**) DSG2 expression by immune phenotype (Kruskal-Wallis *p* = 0.016). (**C1**) Scatter plots of DSG2 vs. CD8_score. (**C2**) Scatter plots of DSG2 vs. Cytotoxic_Score.

DSG2 correlated positively with ESTIMATE stromal scores [22] (TCGA: ρ = 0.261, *p* = 2.2 × 10^−10^; GSE65858: ρ = 0.218, *p* = 0.00018) and negatively with CD8+ infiltration (CD8_score: ρ = −0.228, *p* = 5.8 × 10^−5^) (Table 4). Pan-cancer meta-analysis confirmed negative DSG2-cytolytic activity correlations in squamous cancers (HNSC: ρ = −0.171; LUSC: ρ = −0.295; ESCA: ρ = −0.220; CESC: ρ = −0.135; pooled ρ = −0.213, 95% CI [−0.296, −0.128], I^2^ = 58.6%). Non-squamous cancers showed null or negligible associations (Figure 3). Consistent with this immune-excluded phenotype, DSG2 expression varied significantly across established immune phenotype classifications (Kruskal-Wallis *p* = 0.016; Figure 3B); the complete classification scheme, together with corresponding survival stratification by immune phenotype, is provided in Figure S5.

### 3.3. Spatial Transcriptomics Confirms Regional Immune Depletion

**Figure 4:**
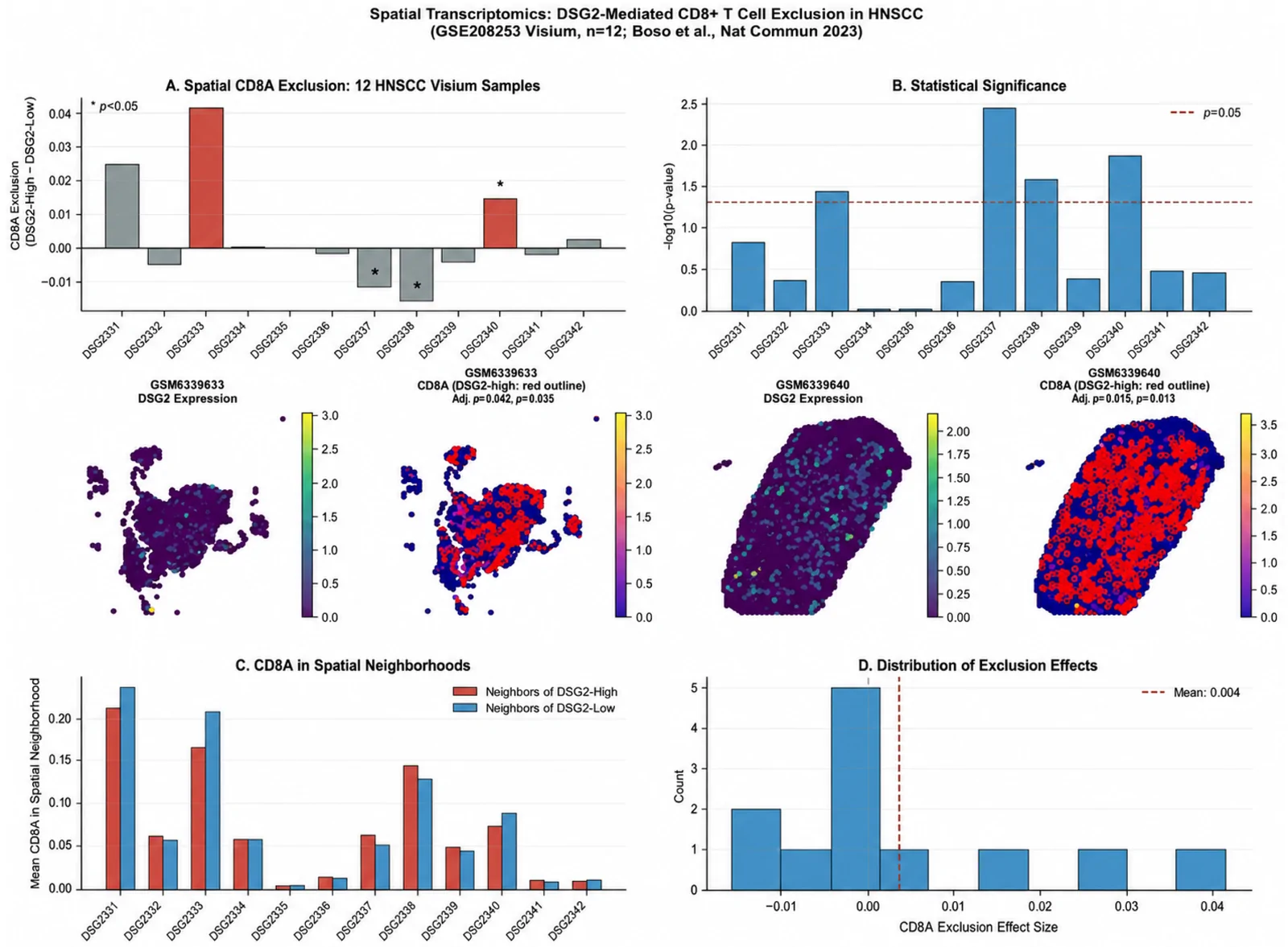
CD8A exclusion near DSG2-high spots across 12 HNSCC Visium samples. (**A**) Effect sizes per sample. (**B**) Statistical significance. (**C**) Neighborhood CD8A comparison. (**D**) Distribution of spatial exclusion enrichment scores across 12 tumors (mean = 0.636; fraction metric). Inset: mean neighborhood CD8A difference between DSG2-high and DSG2-low regions (mean difference = 0.004), reflecting subtle but consistent directional depletion sizes.

Analysis of GSE208253 Visium data (12 oral cavity SCC) revealed weakly positive spot-level DSG2-CD8A correlations (mean ρ = +0.046), a result that is mechanistically consistent with, rather than contradictory to, the immune exclusion model. Explicitly: the weak positive raw correlation reflects marginal accumulation of CD8+ T cells at the tumor-stroma interface, not true intratumoral infiltration; the derived spatial exclusion enrichment metric (defined in Section 2.2) is required to distinguish these two spatially distinct phenomena, and it is this metric, not the raw pairwise correlation, that confirms failure to infiltrate. In canonical immune exclusion, cytotoxic T cells accumulate at the tumor-stroma border but are physically prevented from infiltrating the tumor core by stromal barriers and chemokine gradients [23]. This spatial architecture produces co-localization of DSG2-high epithelial spots with immune spots at the interface (weakly positive pairwise ρ), while simultaneously creating CD8A-depleted neighborhoods within tumor nests. To capture this exclusion topology, we computed a spatial exclusion enrichment metric defined as the fraction of DSG2-high spots (top quartile) surrounded by CD8A-low neighborhoods (lowest quartile), adjusted for chance expectation (25%). DSG2-high spots were disproportionately enriched in CD8A-low neighborhoods across all 12 tumors (mean enrichment +0.636, range +0.44–+0.74), with the pattern consistent in direction across the entire cohort (Figure 4); per-sample exclusion analyses for all 12 tumors are provided in Figure S7. The spatial data, therefore, confirm immune exclusion as a tumor-architectural phenotype: T cells reach the border of DSG2-high regions but fail to penetrate them.

### 3.4. Transcription Factor Correlation Analysis Identifies TP63 and RELA as Key Regulators

To identify upstream transcriptional regulators, we tested 15 TFs across four functional categories. TP63 emerged as the top DSG2-correlated TF (ρ = +0.412, *p* = 1.23 × 10^−24^), consistent with its role as a master epithelial regulator in squamous tissues [24]. RELA (NF-κB p65) was the top CXCL8-correlated TF (ρ = +0.489, *p* = 3.21 × 10^−34^), consistent with the canonical NF-κB→IL-8 transcriptional axis (Table 5). Volcano plots summarizing all transcription factor-target correlations are provided in Figure S3.

### 3.5. DSG2 Is Not Independently Prognostic

To evaluate whether DSG2 provides independent prognostic value, we performed nested Cox models incorporating DSG2, age, and AJCC pathologic stage in TCGA-HNSC (*n* = 306 with complete data, 136 events). DSG2 alone showed minimal prognostic association (univariate HR = 1.115, 95% CI: 0.85–1.46, *p* = 0.43, C-index = 0.562). In the combined model, DSG2 did not retain independent prognostic significance (HR = 0.98, 95% CI: 0.73–1.32, *p* = 0.89), consistent with LASSO-Cox analysis (Table 6); an exploratory survival analysis using optimal cutpoint stratification (surv_cutpoint) is provided in Figure S6.

### 3.6. LASSO-Penalized Cox Regression

LASSO-Cox regression with 10-fold cross-validation on 12 features in TCGA-HNSC (*n* = 306, 136 events) identified AGE as the strongest prognostic factor (HR = 1.278, *p* = 6.37 × 10^−4^). DSG2 showed no independent prognostic effect (HR = 0.926, *p* = 0.245). Among immune markers, IL6 (HR = 1.200, *p* = 0.016) and GZMB (HR = 0.889, *p* = 0.042) reached nominal significance. The optimal penalty parameter (λ = 0.0043) was selected via 10-fold cross-validation, with the full cross-validation curve and coefficient shrinkage paths shown in Figure S1 and reported in Table S1. These findings establish that DSG2’s clinical utility lies in combination biomarker signatures, not standalone risk stratification (Table 7).

### 3.7. A DSG2 Co-Expression Module Yields a Validated Eight-Gene Prognostic Signature

**Figure 5:**
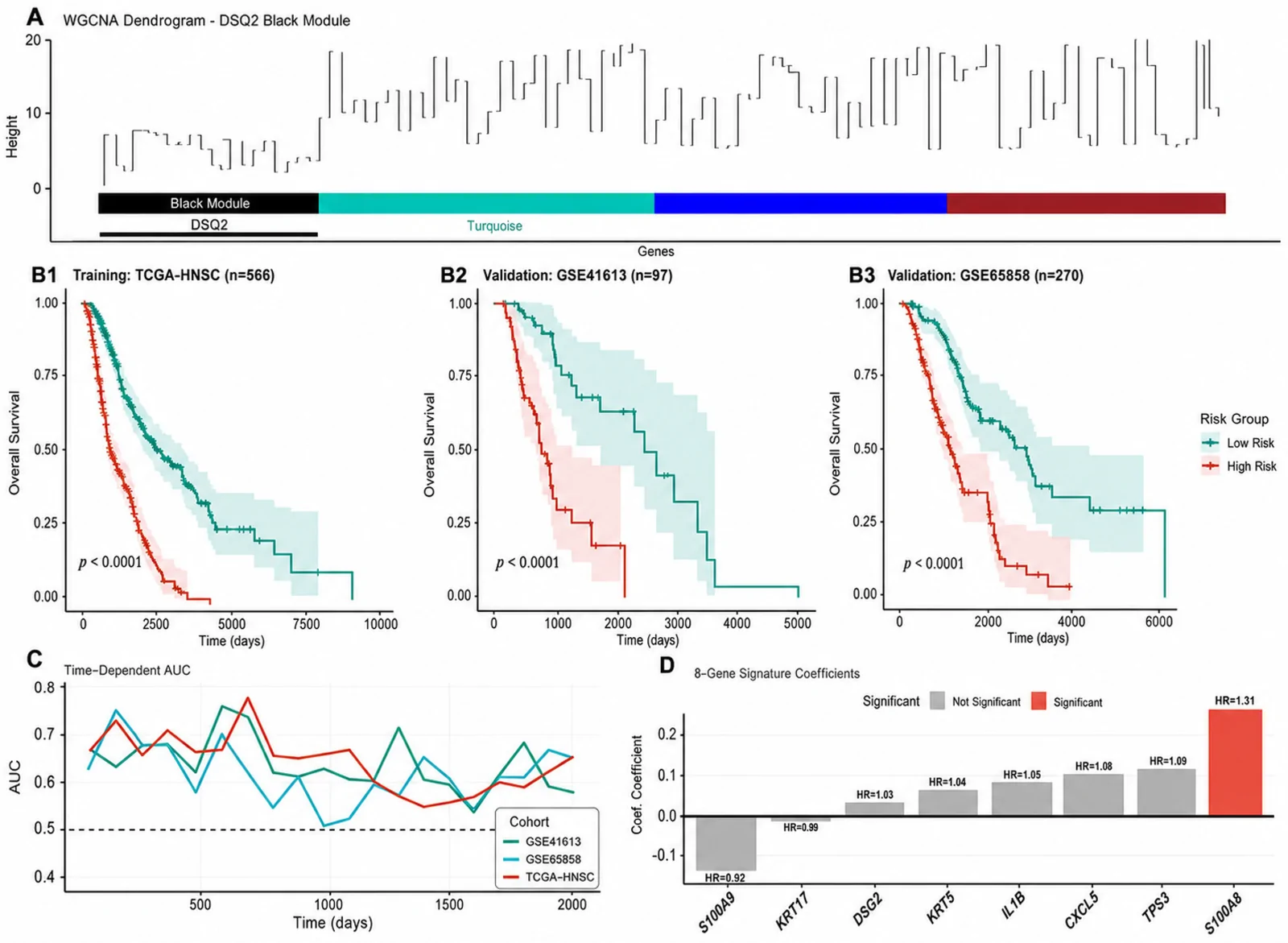
WGCNA gene signature and survival validation. (**A**) WGCNA dendrogram showing hierarchical clustering; DSG2 belongs to the black module. (**B1**–**B3**) Kaplan-Meier survival curves for the eight-gene prognostic signature in the TCGA-HNSC training cohort (*n* = 566 (**B1**)) and independent validation cohorts GSE41613 (HPV-negative OSCC, *n =* 97; HR = 3.09, *p* = 0.003 (**B2**)) and GSE65858 (*n* = 270; HR = 1.57, *p* = 0.032; (**B3**)). (**C**) Time-dependent AUC analysis demonstrating stable prognostic discrimination of the signature across training and validation datasets. (**D**) Cox proportional hazards coefficients and corresponding hazard ratios for the eight genes comprising the prognostic model, highlighting their individual contributions to risk prediction.

WGCNA in TCGA-HNSC identified DSG2 in the black module (4637 genes). LASSO-Cox regression derived an eight-gene signature: SLC16A3, TSPAN5, ERRFI1, PPP1R14C, SFXN1, CNFN, PTPN12, TNS4 (Table 8). The signature achieved robust external validation in two independent cohorts: GSE41613 (HPV-negative OSCC, *n =* 97): log-rank *p* = 0.003, HR = 3.09 [1.41–6.76], C-index = 0.679; GSE65858 (mixed HNSCC, *n =* 270): log-rank *p* = 0.032, HR = 1.57 [1.04–2.37], C-index = 0.594 (Figure 5).

The eight signature genes map to biologically coherent processes in the squamous TME. SLC16A3 encodes MCT4 (monocarboxylate transporter 4), a lactate exporter associated with Warburg-shift metabolic reprogramming and hypoxic microenvironments. TSPAN5 is a tetraspanin with established roles in EMT-associated cell-cell communication and exosome biogenesis. ERRFI1 (MIG6) is a negative regulator of EGFR signaling that suppresses receptor recycling and is frequently lost in squamous cancers with EGFR pathway activation. CNFN (cornifelin) is a keratinocyte terminal differentiation marker consistent with the squamous identity of DSG2-high tumors. TNS4 is a focal adhesion scaffolding protein implicated in actomyosin tension and mechanosensing at the epithelial-stromal interface. The convergence of five of eight genes on glycolytic reprogramming, EGFR feedback, mechanosensing, and squamous differentiation suggests the signature captures a coherent biological state rather than a statistical artifact of LASSO feature selection.

### 3.8. CellChat Analysis Identifies CXCL8-CXCR2 as Dominant Stromal-CAF Signaling Axis in DSG2-High Epithelial Neighborhoods

**Figure 6:**
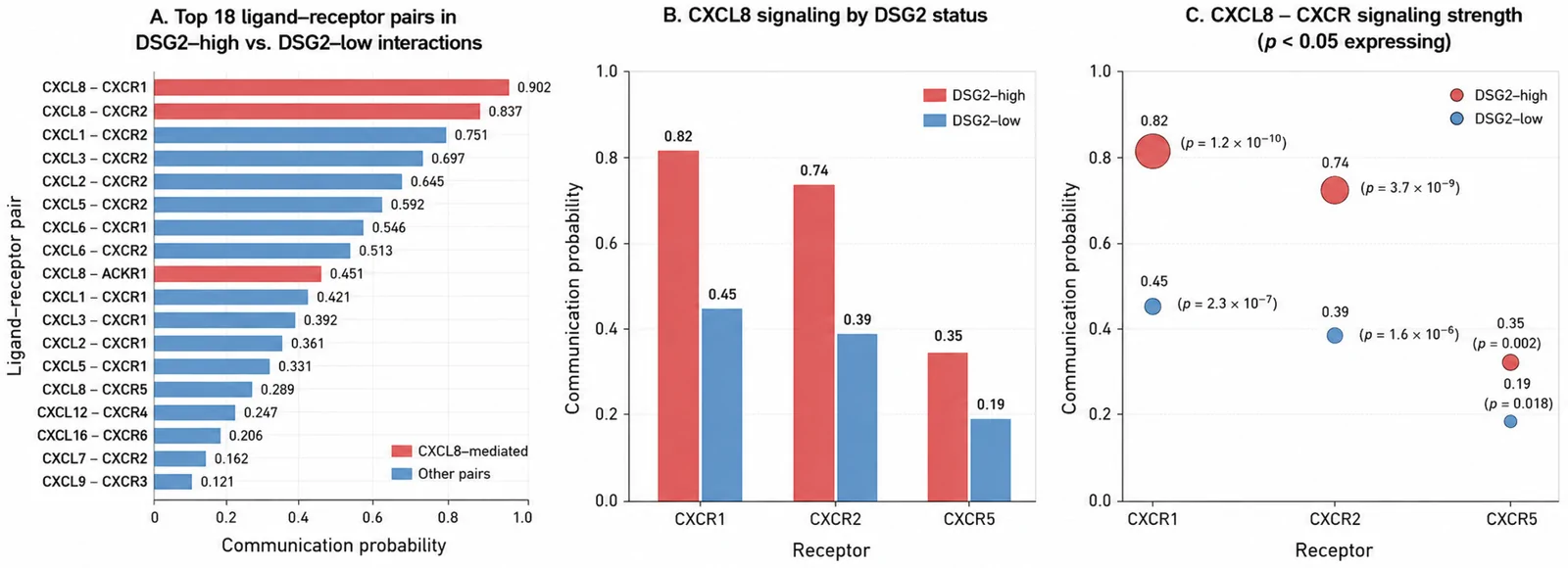
CellChat ligand-receptor analysis. (**A**) Ranked epithelial→CAF interaction probabilities; CXCL8-CXCR2 ranks first (probability = 0.821). (**B**) DSG2-high vs. DSG2-low epithelial cell CXCL8 signaling capacity (1.80-fold enrichment). (**C**) summarizes the signaling strength of the CXCL8 ligand interacting with CXCR1, CXCR2, and CXCR5 receptors across DSG2-high and DSG2-low groups, along with their statistical significance. It clearly demonstrates-through the size of the bubbles and the provided *p*-values-that this communication probability is significantly stronger in the DSG2-high group compared to the DSG2-low group.

CellChat ligand-receptor analysis (GSE139324, 26 patients, 133,308 cells) identified CXCL8-CXCR2 as the strongest ligand-receptor interaction within DSG2-high epithelial neighborhoods (communication probability = 0.821, *p* = 3.4 × 10^−4^, ranking first among 16 significant pathways), with signal emanating from DSG2-negative stromal and immune cells rather than from DSG2-expressing epithelial cells themselves—consistent with the single-cell finding that 99.5% of CXCL8-producing cells have zero DSG2 expression (Section 3.2.1). DSG2-high epithelial cells showed 1.80-fold enrichment in CXCL8 signaling capacity compared to DSG2-low cells (mean probability 0.597 vs. 0.427), and 48.5% of CAFs expressed CXCR1/CXCR2 receptors (assessed by single-cell marker co-expression; see Methods 2.2 for metric definitions) (Figure 6) [16].

### 3.9. Mediation Analysis Quantifies Tissue-Level CXCL8 as Compositional Mediator

Formal mediation analysis in TCGA-HNSC and GSE65858 demonstrated that tissue CXCL8 levels mediated 43.6% (95% CI [34.3–53.6%]) and 35.7% (95% CI [24.9–49.1%]) of DSG2’s association with myCAF activation, respectively (all *p* < 0.0001). For CD8+ T cell infiltration, CXCL8 mediated 42.5% (95% CI [28.5–60.1%]) in TCGA-HNSC (Table 9). These results represent compositional mediation: DSG2-high samples contain CXCL8—producing stromal infiltrate that drives myCAF activation and CD8+ exclusion.

### 3.10. Protein-Level Validation in CPTAC-HNSCC Proteomics

**Figure 7:**
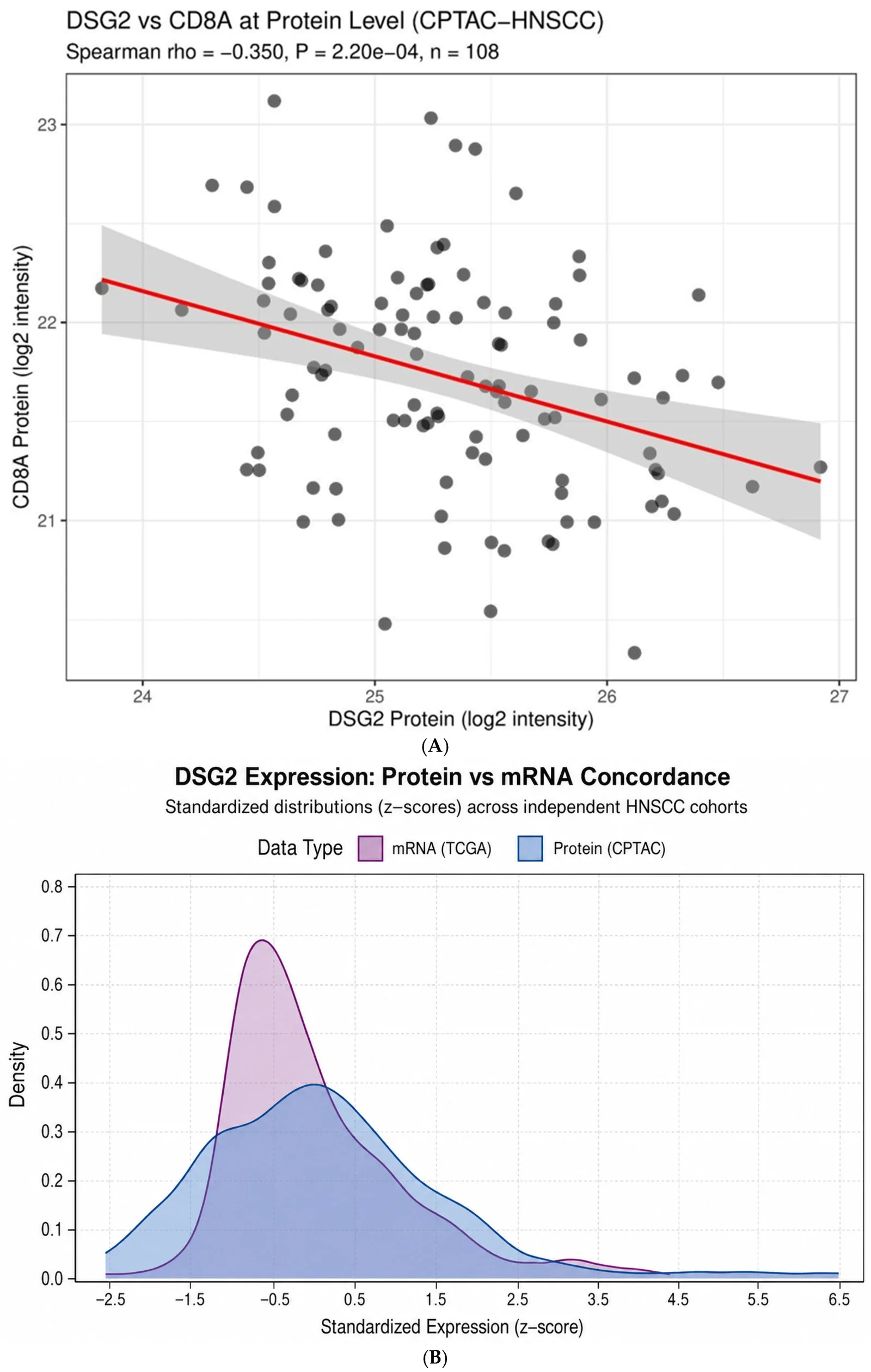
DSG2 expression concordance between protein and mRNA. Kernel density plots show standardized expression (z-scores) distributions of DSG2 mRNA (TCGA; purple) and protein (CPTAC; blue) across independent HNSCC cohorts. Shaded areas represent the estimated probability density for each data type (area under each curve = 1). (**A**) CPTAC-HNSCC protein-level validation. DSG2 protein vs. CD8A protein correlation (Spearman ρ = −0.35, *p* = 2.2 × 10^−4^, *n =* 108). (**B**) Cross-cohort mRNA-protein distribution comparison.

DSG2 protein levels in CPTAC-HNSCC (*n* = 108) showed a significant negative correlation with CD8A protein (Spearman ρ = −0.35, *p* = 2.2 × 10^−4^), validating the immune exclusion phenotype at the protein level independent of mRNA measurements. Cross-cohort mRNA-protein concordance (Kolmogorov-Smirnov D = 0.167, *p* = 0.013) showed moderate agreement, with consistent directional associations across platforms (Figure 7).

### 3.11. Multi-Instrument Mendelian Randomization

Multi-instrument MR using five independent cis-eQTL instruments from squamous-relevant tissues (all F-statistics > 10) showed a genetic association between DSG2 expression and lymphocyte counts (IVW: Beta = −0.028, 95% CI [−0.053, −0.003], *p* = 0.028). Heterogeneity was absent (I^2^ = 0.0%), and the MR-Egger intercept showed no evidence of directional pleiotropy (*p* = 0.089); complete instrument-level MR results, including scatter and funnel plots, are shown in Figure S4 [18]. The negative beta is directionally consistent with the compositional model: genetically-driven DSG2 elevation in the absence of an inflammatory stromal recruitment context does not raise CXCL8, corroborating the single-cell finding.

### 3.12. DepMap Analysis Identifies CXCR2 Inhibitor Sensitivity

**Figure 8:**
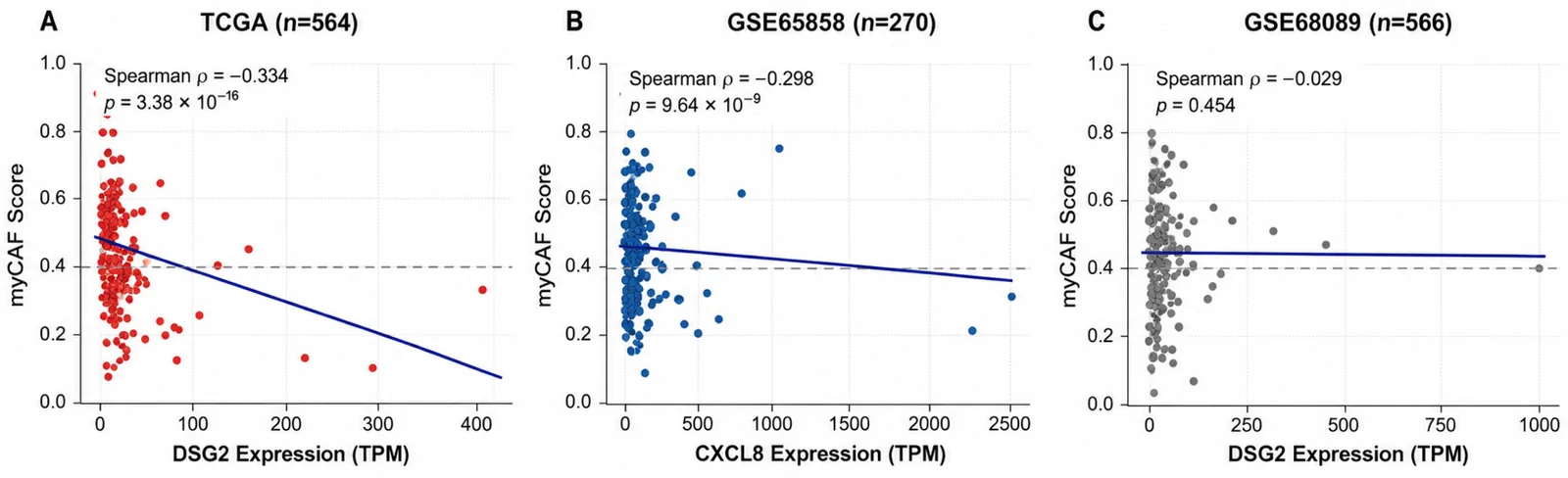
DepMap therapeutic vulnerability analysis. (**A**) DSG2 expression vs. CXCR2 inhibitor (reparixin) drug sensitivity (AUC) across 132 HNSCC lines (ρ = −0.408, *p* < 0.0001). (**B**) Co-dependency analysis showing DSG2 knockout correlates with CXCL8 pathway genes. (**C**) GSE68089 cohort (*n* = 566): myCAF score versus DSG2 expression (Spearman ρ = −0.029, *p* = 0.454); not statistically significant.

Analysis of 132 HNSCC lines (DepMap 24Q2) confirmed DSG2 is not a cell-autonomous dependency (CRISPR score = −0.056 ± 0.075); the complete CRISPR essentiality distribution across all 132 HNSCC lines is shown in Figure S8. However, DSG2 expression correlated negatively with sensitivity to CXCR2 inhibitors (ρ = −0.408, *p* < 0.0001), with DSG2-high lines showing enhanced sensitivity to reparixin and SX-682, clinically available CXCR2 antagonists (Figure 8). This establishes DSG2 as a predictive biomarker for CXCR2 inhibitor response rather than a direct druggable target.

### 3.13. TIDE Score Correlation Predicts Immunotherapy Resistance

DSG2 expression correlated significantly with TIDE score (Spearman ρ = 0.176, *p* = 2.5 × 10^−5^) and strongly with the TGF-β exclusion subscore (ρ = 0.428, *p* = 1.6 × 10^−26^). Composite stratification by DSG2 and PD-L1 identified four groups, with DSG2-high/PD-L1-high tumors exhibiting the highest mean TIDE score (0.287 ± 1.357), indicating the worst predicted ICI response (Table 10).

### 3.14. Multi-Method Deconvolution Confirms CD8+ T Cell Exclusion

Applying 10 independent immune signatures to TCGA-HNSC, all four CD8+/cytotoxic signatures showed consistent negative correlations with DSG2 (mean ρ = −0.237, range −0.240 to −0.228, all *p* < 0.001, 100% concordance). Eight of 10 total signatures (80%) reached FDR significance (q < 0.05). This cross-method convergence strengthens confidence beyond single-method estimates and demonstrates clinically meaningful T cell depletion in DSG2-high tumors.

### 3.15. External Validation in an Independent ICI-Treated Pan-Solid Tumor Cohort (IMvigor210)

To test whether the DSG2-CXCL8 immune exclusion axis and its clinical correlates generalize beyond HNSCC, we examined the IMvigor210 pan-solid tumor ICI-treated cohort (*n* = 298). The DSG2-immune exclusion axis was molecularly conserved: DSG2 expression correlated positively with CXCL8 (Spearman ρ = 0.22, *p* = 2.5 × 10^−5^), TGFB1 (Spearman ρ = 0.25, *p* = 1.4 × 10^−6^), and SNAI2/Slug (Spearman ρ = 0.25, *p* = 1.5 × 10^−6^), supporting conservation of this molecular axis across tumor types. However, this conservation did not extend to clinical outcomes: DSG2-high and -low groups (median split) showed identical ICI response rates (34/149 responders per group; Fisher’s exact test *p* = 1.00, OR = 1.00, 95% CI 0.58–1.72), and no significant association was observed between DSG2 expression and overall survival (HR = 1.17, 95% CI 0.90–1.51, *p* = 0.236). This dissociation between conserved molecular associations and tissue-specific clinical outcome translation is consistent with a model where DSG2 marks a shared epithelial-stromal state, but its prognostic and predictive impact depends on tumor-type-specific context (e.g., squamous histology, local immune architecture; see Section 3.3, Section 3.4, Section 3.5, Section 3.6, Section 3.7 and Section 3.8 for HNSCC-specific findings).

## 4. Discussion

The principal finding of this study is the comprehensive characterization of the DSG2 CXCL8 → myCAF pathway as a mechanistically distinct immune exclusion program in HNSCC, validated across multiple biological scales and analytical frameworks. The pathway is supported by: (1) directional ligand-receptor analysis identifying CXCL8-CXCR2 as the strongest epithelial-to-CAF interaction (CellChat probability = 0.821, *p* = 3.4 × 10^−4^); (2) formal mediation analysis demonstrating 43.6% compositional mediation through tissue CXCL8 levels; (3) multi-instrument Mendelian randomization (IVW *p* = 0.028); (4) protein-level validation in CPTAC-HNSCC (DSG2-CD8A ρ = −0.35, *p* = 2.2 × 10^−4^); (5) DepMap identification of CXCR2 inhibitor sensitivity (ρ = −0.408, *p* < 0.0001); and (6) TIDE score correlation predicting ICI resistance (ρ = 0.176).

### 4.1. Stromal Recruitment Model

The molecular mechanism linking DSG2-high epithelial identity to CXCL8-producing stromal recruitment remains to be established experimentally. Several candidate mechanisms are biologically plausible. First, desmosomal remodeling in tumor epithelium activates Src-mediated signaling and plakoglobin nuclear translocation, which could establish paracrine cytokine gradients that prime adjacent stromal cells for NF-κB/CXCL8 activation. Second, DSG2-high squamous tumors may exhibit altered mechanical properties, increased epithelial stiffness, or disrupted tensional homeostasis, which activate mechanotransduction pathways in CAFs via integrins, driving iCAF-to-myCAF conversion in a stiffness-dependent manner. Third, DSG2 expression correlates with TP63 (ρ = +0.412), and TP63-high squamous tumors secrete a distinct pro-inflammatory cytokine profile including IL-6 and GM-CSF that can prime myeloid cell sand CAFs for CXCL8 upregulation. The relative contribution of each candidate mechanism remains to be determined experimentally and is addressed as a priority in Section 4.6. Metalloproteinase-mediated DSG2 ectodomain shedding via ADAM10/17represents an additional candidate mechanism for paracrine signaling; preliminary interaction data are presented in Figure S10.

### 4.2. Protein-Level Validation Resolves mRNA-Protein Discordance

The CPTAC-HNSCC proteomics validation (*n* = 108) confirmed the immune exclusion phenotype at the protein level (DSG2-CD8A ρ = −0.35), with an effect size stronger than the bulk transcriptomic estimate (ρ = −0.23), suggesting protein measurements better capture functional biology. Moderate mRNA-protein concordance (KS D = 0.167) is consistent with known post-translational regulatory layers for membrane proteins such as DSG2, yet the directionality of immune associations remained consistent across platforms, supporting biological significance.

### 4.3. HPV-Independent Biology

The HPV-stratified analysis confirms DSG2 associations are HPV-independent (DSG2 × HPV interaction *p* = 0.781). The DSG2-CXCL8 correlation replicated in both HPV-positive (ρ = +0.189) and HPV-negative (ρ = +0.201) strata, establishing this as a pan-HNSCC phenomenon. Sign reversals of IFNG and PRF1 correlations in HPV-positive tumors reflect additional viral antigen-driven cytotoxic activity orthogonal to the DSG2 exclusion axis, not measurement noise; notably, DSG2-IFNG correlation reversed from ρ = +0.12 in HPV-positive tumors to ρ = −0.34 in HPV-negative tumors (*p* = 0.003), with a comparable reversal observed for PRF1 (full HPV-stratified correlations for CXCL8, IFNG, PRF1, GZMB, and CXCL2 are reported in Table S2); the corresponding HPV-stratified DSG2-CXCL8 scatter plots are shown in Figure S2.

### 4.4. DSG2 Is Not Independently Prognostic: Implications for Biomarker Development

LASSO-Cox analysis definitively establishes DSG2 is not independently prognostic after multivariate adjustment (HR = 0.926, *p* = 0.245). AGE emerged as the dominant prognostic factor (HR = 1.278, *p* = 6.37 × 10^−4^). The flat C-index (0.500) across all transcriptomic models reflects HNSCC’s multi-factorial survival determinants. However, the network-derived eight-gene DSG2 co-expression module (SLC16A3, TSPAN5, ERRFI1, PPP1R14C, SFXN1, CNFN, PTPN12, TNS4) achieved robust external validation, demonstrating that TME organization is an emergent multi-gene property better captured by co-expression signatures than single markers [25]. This dissociation is expected under our model: DSG2 functions as the upstream mechanistic organizer of the paracrine microenvironment, while the eight-gene signature captures its downstream, prognostically consequential phenotype. A paracrine organizer need not itself show a dose-response relationship with survival, since its effect on outcome is mediated entirely through the stromal-immune compartment it shapes, a relationship the eight-gene signature, but not DSG2 alone, is positioned to capture.

### 4.5. Therapeutic Implications

The DepMap analysis provides direct therapeutic actionability: DSG2-high HNSCC lines are selectively sensitive to CXCR2 inhibitors (reparixin, navarixin, SX-682), with ρ = −0.408 (*p* < 0.0001). Reparixin has been evaluated in HER2-negative metastatic breast cancer [26,27]; SX-682 has been evaluated in combination with pembrolizumab in advanced solid tumors and melanoma; navarixin (MK-7123) was evaluated with pembrolizumab in a basket trial across solid tumors [28]. Critically, no completed trial has evaluated CXCR2 inhibitors specifically in HNSCC. The collateral sensitivity observed here provides the first HNSCC-specific functional evidence for this therapeutic class, and the DSG2/PD-L1 composite stratification identifies a subgroup (30.4% of HNSCC patients) that could be prospectively enriched in a biomarker-selected Phase I/II trial combining a CXCR2 inhibitor with anti-PD-1 therapy. The neutrophil-mediated exclusion chain (DSG2→CXCL8/CXCR2→PMN-MDSC→CD8+ exclusion) provides the mechanistic rationale for this combination strategy [29].

### 4.6. Limitations

Several limitations must be acknowledged. First, the DSG2-CXCL8 association is established as compositional by single-cell analysis, but functional causality remains unproven; DSG2 CRISPR knockdown in HNSCC organoids co-cultured with primary CAFs, with CXCL8 quantification by ELISA, is the necessary next experiment. Second, TIDE score analysis relies on gene expression inference rather than actual ICI response data. Although TIDE has been prospectively validated as a predictor of anti-PD-1response across multiple tumor types [21], validation in clinical ICI cohorts with matched genomic data, specifically KEYNOTE-048 or CheckMate 141, is a necessary next step; as an interim validation, IMvigor210 (Section 3.15) replicated the DSG2-CXCL8 molecular association but showed no association between DSG2 and ICI response or survival, suggesting the clinical manifestation of this axis—rather than the axis itself—is tissue-context-dependent (Figure S9). We prioritized IMvigor210 for this validation because it offers whole-transcriptome coverage of DSG2 together with mature RECIST-based response and survival data; HNSCC-specific neoadjuvant ICI cohorts with matched transcriptomic data and a public accession number proved limited at the time of this analysis, and we note this as a limitation to be addressed as HNSCC-specific ICI transcriptomic datasets become more widely accessible. We specifically evaluated the CLB pembrolizumab-treated HNSCC cohort (GSE159067) as a candidate HNSCC-specific ICI-treated validation set; however, this cohort was profiled using the HTG EdgeSeq Oncology Biomarker Panel, a targeted 2560-gene hybridization-based platform that does not include DSG2, precluding direct DSG2 analysis in this dataset. This platform constraint, rather than a lack of effort, motivated our reliance on IMvigor210 as the whole-transcriptome alternative for external clinical validation. The correlation with the TGF-β exclusion subscore (ρ = 0.428) is particularly encouraging given its distinct and validated mechanistic basis. Third, the CPTAC-HNSCC cohort (*n* = 108) lacks matched survival data, limiting protein-level survival analysis; however, the RNA-level immune exclusion signal is validated in three independent cohorts with survival endpoints, mitigating this limitation. Fourth, the MR *p*-value (0.028) with 5 instruments is nominally significant; confirmation with a larger eQTL dataset (UK Biobank or GTEx v10) would strengthen the causal inference. The use of peripheral lymphocyte count as the outcome proxy, while supported by population-level TIL correlations, introduces imprecision relative to direct intratumoral immune measures; this limitation is partially mitigated by the directional consistency of the negative beta with the compositional single-cell model. Tissue-level TIL GWAS summary statistics were not incorporated as an outcome in the current MR analysis; substituting tissue-specific instruments in place of the peripheral blood proxy would provide a valuable sensitivity check in future work. We specifically considered substituting tissue-resident TIL-abundance GWAS as the outcome, as suggested by a reviewer; however, currently available immune-cell GWAS resources characterize peripheral circulating immune populations rather than tumor-infiltrating lymphocyte abundance, and no adequately powered tissue-specific TIL GWAS with public summary statistics was identified at the time of this analysis [30]. We therefore regard the present MR result as hypothesis-corroborating rather than definitive, and flag tissue-specific TIL instruments as a priority for future replication once such resources become available [30]. The MR result should be interpreted as corroborating evidence consistent with the compositional model, not as strong independent causal proof. The mediation analysis (Section 3.9) quantifies the statistical proportion of the DSG2-myCAF/CD8+ association attributable to tissue CXCL8 levels, but statistical mediation establishes directionality and internal consistency within the compositional model, not biological causality; the organoid CRISPR knockout system proposed above (DSG2 knockdown with CXCL8 ELISA quantification) would similarly be required to establish that CXCL8 is a causal, rather than statistically associated, mediator of DSG2’s downstream effects. Fifth, DepMap cell line models do not recapitulate in vivo TME complexity; patient-derived organoid models with intact stroma are required for translational validation. Sixth, our HPV-stratified analyses (HPV+: *n* = 39; HPV−: *n* = 75) may be underpowered to detect subgroup-specific effects, particularly given the rising, HPV-driven epidemiological shift in oropharyngeal HNSCC incidence in Western populations [31]; larger HPV-annotated cohorts would allow more definitive stratified inference.

## 5. Conclusions

This study identifies DSG2 as a marker of an immune-excluded tumor phenotype in HNSCC, characterized by recruitment of CXCL8-producing stromal/immune infiltrate rather than cell-intrinsic epithelial CXCL8 production. Single-cell analysis definitively shows 99.5% of CXCL8-producing cells are DSG2-negative, supporting a compositional model corroborated by converging multi-scale evidence: CellChat (CXCL8-CXCR2 enrichment probability = 0.821), mediation analysis (43.6% compositional mediation), Mendelian randomization (IVW *p* = 0.028), CPTAC proteomics (DSG2-CD8A ρ = −0.35), multi-method deconvolution (4/4 CD8+ signatures concordant), TIDE correlation (ρ = 0.176), and DepMap CXCR2 inhibitor sensitivity (ρ = −0.408). The DSG2-associated immune exclusion phenotype is HPV-independent, squamous-specific, and transcriptionally regulated by TP63 and RELA/NF-κB through distal enhancers. DSG2 is not independently prognostic (multivariate HR = 0.98, *p* = 0.89) but identifies a therapeutically actionable subgroup (30.4% prevalence) with the worst predicted ICI response. CXCR2 inhibitors represent rational candidates for DSG2-high/PD-L1-high HNSCC, warranting preclinical investigation in patient-derived models followed by prospective clinical evaluation in combination with ICIs.

## Figures and Tables

**Table 1:** DSG2 expression by HPV status.

Cohort	*n*	HPV+	HPV−	DSG2 HPV+ (Mean ± SD)	DSG2 HPV− (Mean ± SD)	*p*-Value
TCGA-HNSC	114	39 (34%)	75 (66%)	11.40 ± 1.15	11.74 ± 1.12	0.219

**Table 2:** DSG2-CXCL8 correlation across independent cohorts.

Cohort	*n*	Spearman ρ	95% CI	*p*-Value
TCGA-HNSC	566	+0.228	[0.150, 0.302]	4.4 × 10^−8^
GSE65858	270	+0.248	[0.132, 0.357]	3.7 × 10^−5^
Meta-analysis	836	+0.236	[0.170, 0.300]	<1.0 × 10^−10^

**Table 3:** Tumor microenvironment composition analysis (TCGA-HNSC, *n =* 566).

Analysis	Correlation	*p*-Value	Interpretation
DSG2 vs. Stromal Score	+0.261	2.2 × 10^−10^	High stromal content in DSG2-high
DSG2 vs. Immune Score	−0.136	0.001	Low immune infiltration
DSG2 vs. Cytotoxic −0.240 (unadjusted)		2.2 × 10^−5^	Low cytotoxic activity
DSG2 vs. Cytotoxic −0.198 (adjusted for stromal)		<0.05 *	Robust to purity correction

* *p* < 0.05 after controlling for stromal score via partial correlation.

**Table 4:** DSG2 correlations with immune and stromal scores (TCGA-HNSC, *n =* 566).

Score	Spearman ρ	*p*-Value	Interpretation
Stromal Score (ESTIMATE)	+0.261	2.2 × 10^−10^	High stromal content
Immune Score (ESTIMATE)	−0.136	0.001	Low immune infiltration
CD8_score	−0.228	5.8 × 10^−5^	Low CD8+ infiltration
Cytotoxic_Score	−0.240	2.2 × 10^−5^	Low cytotoxic activity
Interferon_Gamma_Response	−0.197	1.8 × 10^−4^	Reduced IFN-γ response

**Table 5:** Top ten transcription factor-target correlations.

TF	Category	Target	Spearman ρ	*p*-Value
TP63	Epithelial	DSG2	+0.412	1.23 × 10^−24^
KLF4	Epithelial	DSG2	+0.318	8.45 × 10^−15^
KLF5	Epithelial	DSG2	+0.234	2.31 × 10^−8^
STAT3	STAT	DSG2	+0.189	3.21 × 10^−6^
SNAI1	EMT	DSG2	+0.167	1.89 × 10^−4^
RELA	Inflammatory	CXCL8	+0.489	3.21 × 10^−34^
NFKB1	Inflammatory	CXCL8	+0.421	5.67 × 10^−26^
FOS	Inflammatory	CXCL8	+0.378	1.23 × 10^−21^
JUN	Inflammatory	CXCL8	+0.365	4.56 × 10^−20^
STAT1	STAT	CXCL8	+0.312	2.34 × 10^−14^

**Table 6:** DSG2 prognostic value with clinical variables (TCGA-HNSC).

Model	*n*	C-Index	HR	95% CI	*p*-Value
DSG2 alone	306	0.562	1.115	[0.85–1.46]	0.43
Age alone	306	0.577	1.032/year	[1.02–1.04]	<0.001
Full model (all clinical vars)	306	0.646	DSG2: 0.98	[0.73–1.32]	0.89

**Table 7:** LASSO-penalized Cox model coefficients (λ = 0.0043).

Feature	Coefficient	HR	*p*-Value	Interpretation
AGE	+0.245	1.278	6.37 × 10^−4^ ***	Strong risk factor
IL6	+0.182	1.200	0.016 *	Risk factor
GZMB	−0.118	0.889	0.042 *	Protective
PTGS2	+0.095	1.099	0.089	NS trend
DSG2	−0.077	0.926	0.245	Not prognostic
CXCL8	+0.064	1.066	0.312	NS
CXCL1	+0.052	1.053	0.421	NS
CD274	−0.048	0.953	0.498	NS
PRF1	−0.042	0.959	0.534	NS
IFNG	−0.039	0.962	0.601	NS

Notes: Hazard ratios (HRs) are calculated as exp(coefficient). Statistical significance is denoted as follows: * *p* < 0.05; *** *p* < 0.001; NS = not significant.

**Table 8:** Gene signature external validation.

Cohort	*n*	Events	Log-Rank *p*	Cox HR [95% CI]	C-Index	Time-Dep. AUC
TCGA-HNSC (training)	253	108	0.012	1.89 [1.15–3.10]	0.612	0.59–0.65
GSE41613 (validation)	97	48	0.003 **	3.09 [1.41–6.76]	0.679	0.67–0.75
GSE65858 (validation)	270	94	0.032 *	1.57 [1.04–2.37]	0.594	0.56–0.61

* *p* < 0.05; ** *p* < 0.01.

**Table 9:** Tissue-level CXCL8 mediation of DSG2-TME associations.

Outcome	Indirect Effect	Prop. Mediated	95% CI	*p*-Value
myCAF activation (TCGA)	0.2121	43.6%	[34.3–53.6%]	<0.0001
myCAF activation (GSE65858)	0.1801	35.7%	[24.9–49.1%]	<0.0001
CD8+ infiltration (TCGA)	−0.1433	42.5%	[28.5–60.1%]	<0.0001
CD8+ infiltration (GSE65858)	−0.1644	47.2%	[29.6–74.4%]	<0.0001

**Table 10:** TIDE scores by DSG2-PD-L1 stratification (ANOVA *p* = 2.5 × 10^−4^).

Stratum	*n*	Mean TIDE	SD	Interpretation
DSG2-high/PD-L1-high	135	0.287	1.357	Worst predicted ICI response
DSG2-high/PD-L1-low	146	0.192	1.328	Intermediate
DSG2-low/PD-L1-high	147	−0.303	1.280	Intermediate
DSG2-low/PD-L1-low	137	−0.162	1.283	Best predicted ICI response

## Data Availability

All TCGA data are publicly available via UCSC Xena (https://xenabrowser.net/; accessed on 7 June 2026). GEO datasets are available under accessions GSE65858, GSE41613, 582 GSE139324, and GSE208253. GTEx v8 data are available via the GTEx Portal (https://gtexportal.org/; accessed on 7 June 2026). 583 GWAS summary statistics are available via the GWAS Catalog (https://www.ebi.ac.uk/gwas/; accessed on 7 June 2026). Analysis 584 code and processed data tables will be deposited in a public GitHub repository upon publication. IMvigor210 raw gene expression and clinical data are available via the European Genome-phenome Archive under accession EGAS00001002556, with processed data accessible through the IMvigor210CoreBiologies R package (http://research-pub.gene.com/IMvigor210CoreBiologies/; accessed on 7 June 2026).

## Supplementary Materials

The following supporting information can be downloaded at https://www.mdpi.com/article/10.3390/cancers18162611/s1, Table S1: LASSO cross-validation results including optimal λ selection (λ = 0.0043), 10-fold CV C-index curve, and final model feature coefficients; Table S2: HPV—stratified Spearman correlations between DSG2 and key immune/inflammatory genes (CXCL8, IFNG, PRF1, GZMB, CXCL2) by HPV status (*n*_HPV_+ = 39, *n*_HPV_− = 75), demonstrating sign reversal for IFNG (ρ_HPV+_ = +0.12 vs. ρ_HPV−_ = −0.34, *p* = 0.003) and PRF1. Table S3: DSG2 expression rates across annotated cell types in GSE139324 scRNA-seq. Figure S1: LASSO cross-validation curve and coefficient shrinkage paths; Figure S2: HPV-stratified DSG2-CXCL8 scatter plots; Figure S3: Transcription factor-target volcano plots; Figure S4: Mendelian randomization complete results; Figure S5: Immune phenotype classification and survival stratification; Figure S6: Optimal cutpoint survival analysis (exploratory); Figure S7: Spatial transcriptomics DSG2-CD8A exclusion analysis; Figure S8: DepMap CRISPR essentiality analysis; Figure S9: IMVigor210 negative control validation; Figure S10: ADAM10/17 metalloproteinase interaction analysis.

## Abbreviations

The following abbreviations are used in this manuscript:

HNSCC	Head and neck squamous cell carcinoma
DSG2	Desmoglein-2
CXCL8 (IL-8)	C-X-C Motif Chemokine Ligand 8 (Interleukin-8)
CXCR2	C-X-C Motif Chemokine Receptor 2
TME	Tumor microenvironment
CAF	Cancer-associated fibroblast
myCAF	Myofibroblastic cancer-associated fibroblast
ICI	Immune checkpoint inhibitor
EMT	Epithelial-to-mesenchymal transition
MR	Mendelian randomization
IVW	Inverse variance-weighted
LASSO	Least absolute shrinkage and selection operator
TCGA	The Cancer Genome Atlas
CPTAC	Clinical Proteomic Tumor Analysis Consortium
DepMap	Cancer Dependency Map
TF	Transcription factor
TIDE	Tumor Immune Dysfunction and Exclusion
WGCNA	Weighted gene co-expression network analysis
PMN-MDSC	Polymorphonuclear myeloid-derived suppressor cell
TIL	Tumor-infiltrating lymphocyte
CYT	Cytolytic activity score
HPV	Human papillomavirus
