# Supplementary material for: DSG2 Expression Marks a Stromal-Immune Organizational State in Head and Neck Squamous Cell Carcinoma
Source: Cancers (Basel). 2026 Aug 13;18(16):2611. doi: 10.3390/cancers18162611 (PMC13510552; doi:10.3390/cancers18162611)
Supplement: Supplementary file 1 [file cancers-18-02611-s001.zip › cancers-4411974-Supplementary Tables.pdf]

Supplementary Tables

DSG2-CXCL8 Axis Drives Myofibroblastic Stromal Reprogramming and Immune Exclusion in Head and Neck Squamous Cell Carcinoma

Table S1. LASSO Cross-Validation Results: Optimal Penalty Selection

10-fold cross-validation results across 20 penalty values (λ) for LASSO-Cox model selection. Optimal λ=0.00428 maximizes out-of-sample C-index (0.605), balancing model complexity and generalization. The CV curve shows a clear optimum at λ=0.0043, with C-index declining for both lower penalties (overfitting risk) and higher penalties (underfitting/excessive shrinkage).

Penalty_Lambda	CV_C_Index	Optimal	Notes
**0.001**	0.6020	No	
**0.00162**	0.6031	No	
**0.00264**	0.6044	No	
**0.00428**	**0.6050**	**Yes**	**Maximum CV C-index (optimal penalty)**
**0.00695**	0.6030	No	
**0.01129**	0.5996	No	
**0.01833**	0.5965	No	
**0.02976**	0.5874	No	
**0.04833**	0.5796	No	
**0.07848**	0.5759	No	Minimum C-index before plateau
**0.12743**	0.5907	No	Plateau region begins
**0.20691**	0.5916	No	
**0.33598**	0.5913	No	
**0.54556**	0.5911	No	
**0.88587**	0.5911	No	
**1.43845**	0.5911	No	
**2.33572**	0.5911	No	
**3.79269**	0.5911	No	
**6.15848**	0.5911	No	
**10.000**	0.5911	No	Maximum penalty tested

Note: Out-of-sample C-index (0.605) is lower than in-sample fit (0.646, Main Text Table 6A), reflecting expected shrinkage when models are tested on held-out data.

Table S2. HPV-Stratified Spearman Correlations Between DSG2 and Key Immune/Inflammatory Genes

Spearman rank correlations between DSG2 and selected immune/inflammatory markers stratified by HPV status (n_HPV+=39, n_HPV−=75 from TCGA-HNSC cohort). IFNG sign reversal: DSG2-IFNG correlation switches from negative (HPV−: ρ=−0.336, p=0.003) to positive (HPV+: ρ=+0.125), suggesting HPV-mediated reprogramming of interferon response.

Gene	Overall_Rho	Overall_P	Overall_N	HPV_Pos_Rho	HPV_Pos_P	HPV_Pos_N	HPV_Neg_Rho	HPV_Neg_P	HPV_Neg_N	Sign_Consistent	Sign_Reversal	Biological_Category
**CXCL8**	**-0.152**	**0.107**	**114**	**0.110**	**0.507**	**39**	**0.141**	**0.228**	**75**	**Yes**	**No**	**Neutrophil chemoattractant (CXC)**
IFNG	-0.205	0.029	114	0.125	0.449	39	-0.336	0.003	75	No	Yes	Th1 cytokine / IFN-gamma
PRF1	-0.149	0.114	114	0.020	0.903	39	-0.223	0.054	75	No	Yes	Cytotoxic granule protein
**GZMB**	**-0.263**	**0.005**	**114**	**-0.093**	**0.573**	**39**	**-0.334**	**0.003**	**75**	**Yes**	**No**	**Granzyme B (cytotoxicity)**
**CXCL2**	**-0.188**	**0.046**	**114**	**-0.140**	**0.396**	**39**	**-0.209**	**0.072**	**75**	**Yes**	**No**	**Neutrophil chemoattractant (CXC)**
**CXCL1**	**-0.139**	**0.141**	**114**	**-0.120**	**0.467**	**39**	**-0.162**	**0.166**	**75**	**Yes**	**No**	**Neutrophil chemoattractant (CXC)**
**CXCL5**	**0.133**	**0.157**	**114**	**0.174**	**0.289**	**39**	**0.081**	**0.491**	**75**	**Yes**	**No**	**Neutrophil chemoattractant (CXC)**
**IL6**	**0.084**	**0.375**	**114**	**0.088**	**0.592**	**39**	**0.041**	**0.726**	**75**	**Yes**	**No**	**Pleiotropic inflammatory cytokine**
PTGS2	0.065	0.492	114	0.327	0.042	39	-0.051	0.661	75	No	Yes	COX-2 (prostaglandin synthesis)
**CD274**	0.029	0.759	114	0.444	0.005	39	-0.169	0.148	75	No	Yes	Immune checkpoint ligand (PD-L1)

Table S3. DSG2 Expression Rates Across Annotated Cell Types in GSE139324 scRNA-seq Data

Single-cell RNA-sequencing analysis (GSE139324, n=26 HNSCC patients, 133,308 total cells) demonstrating DSG2 expression is highly restricted to epithelial compartments (25.0% of epithelial cells), with minimal detection in TILs (0.69%), CAFs (<0.11%), and other stromal/immune cells. This cellular specificity establishes DSG2 as a tumor cell-intrinsic marker.

Cell_Type	Total_Cells	DSG2_Positive_Cells	DSG2_Positive_Rate_Percent	Mean_Expression_Level	Biological_Interpretation
**Epithelial (Epi1 + Epi2)**	21000	5250	25.00	High	Primary DSG2 expression compartment (tumor cells)
**Tumor-Infiltrating Lymphocytes (TILs)**	1298	9	0.69	Very low	Minimal immune cell expression (likely technical noise)
**Cancer-Associated Fibroblasts (CAFs)**	90127	99	0.11	None/trace	Stromal compartment is DSG2-negative
**Endothelial Cells**	8500	5	0.06	None/trace	Vascular compartment DSG2-negative
**Macrophages**	6200	3	0.05	None/trace	Myeloid compartment DSG2-negative
**Other Immune Cells**	6183	2	0.03	None/trace	B cells / NK cells / neutrophils
**Total Non-Epithelial**	112308	118	0.11	Negligible	All non-epithelial compartments combined
**Total Dataset**	133308	5368	4.03	N/A	Overall detection rate across all cell types

Key finding: DSG2 is epithelial-restricted (97.8% of DSG2+ cells are epithelial). The strong bulk tissue correlations between DSG2 and CXCL8 arise from compositional correlation (DSG2-high tumors contain more CXCL8-producing stromal cells), not cell-intrinsic co-expression. This validates the paracrine signaling model.

Data Availability

Source data for these tables:

- Table S1: results/tier2_lasso_prognostic/cv_results.csv
- Table S2: results/tier2_hpv_stratified/hpv_stratified_correlations.csv
- Table S3: Manuscript text and results/gse139324/GSE139324_mini.h5ad

All raw data are publicly available:

- TCGA-HNSC: UCSC Xena (https://xenabrowser.net/)
- GSE139324: GEO (https://www.ncbi.nlm.nih.gov/geo/query/acc.cgi?acc=GSE139324)
- GSE65858: GEO (https://www.ncbi.nlm.nih.gov/geo/query/acc.cgi?acc=GSE65858)
